# Guilty by association? SARS‐CoV‐2 antibodies and myelin oligodendrocyte glycoprotein antibody‐associated disease

**DOI:** 10.1111/ene.15335

**Published:** 2022-04-03

**Authors:** Radu Tanasescu, Markus Reindl

**Affiliations:** ^1^ 6123 Academic Clinical Neurology Division of Clinical Neuroscience University of Nottingham Nottingham UK; ^2^ Department of Neurology Nottingham University Hospitals National Health Service Trust Nottingham UK; ^3^ Clinical Department of Neurology Medical University of Innsbruck Innsbruck Austria

It is now well established that many COVID‐19 patients can present with neurological syndromes, although the SARS‐CoV‐2 virus is rarely detected in cerebrospinal fluid or brain tissue. Therefore, it is likely that SARS‐CoV‐2 infections, as with many other viruses, can trigger postinfectious autoimmune diseases [1]. In this issue of *European Journal of Neurology*, Mariotto et al. have analysed serum samples from patients sent for myelin oligodendrocyte glycoprotein (MOG)–immunoglobulin G (IgG) testing for the presence of SARS‐CoV‐2 antibodies, to elucidate whether there is a correlation between COVID‐19 and MOG antibody‐associated disease (MOGAD) [2]. They report that SARS‐CoV‐2 IgG is more common in MOGAD than in controls and suggest that their findings provide preliminary data on the role of SARS‐CoV‐2 infection as a potential trigger of MOGAD.

The study is interesting for a number of reasons. It is the first study to date to look systematically into such a possible correlation in a larger group of MOGAD patients. By the nature of the study, the results raise to another level the debate on COVID‐19 as a trigger for MOGAD, which is at this time still at the stage of case reports [3]. The limitations are several (single centre, short follow‐up, and still too small a sample size to infer a definite conclusion; other potential confounders such as the impact of other viral infections; lack of information on timing of occurrence of MOG antibodies after infection, which is key in discussing causation). Furthermore, the control group is not well selected, because it is not fully matched by clinical presentation and it is unclear how both the COVID‐19 pandemic and increasing awareness of MOGAD among neurologists [4] might have influenced these results.

This study reports, in a group of newly diagnosed people with MOGAD, that SARS‐CoV‐2 IgG was twice more commonly found than in controls. This difference does not pass the threshold for significance of a Fisher test, but with such low numbers, the *p*‐values may not be meaningful. The odds ratio is 2.67, but its 95% confidence interval crosses 1 (0.85–9.17). A power calculation shows the study is underpowered (39% power) and a sample size of at least 81 subjects in each group is needed to reach significance. The main value of this study therefore lies in providing pilot data to calculate estimations of effect sizes, which can allow future studies with a sample size adapted to a proper power. However, another recent study indicated that MOG‐IgG‐seropositive acute disseminated encephalomyelitis associated with SARS‐CoV‐2 infection is a rare finding [5] which should also be considered when planning future epidemiological studies.

The study of Mariotto et al. leaves many questions unanswered. Despite the possible relevant association, things are far from black and white. If possibly triggered, is the MOGAD post‐COVID‐19 more benign? Are these people likely to have a monophasic course? Long‐term follow‐up is mandatory, the more so because it is not always clear that persistent MOG antibodies themselves are always pathogenic. Is there an immunological cross‐reactivity between MOG and SARS‐CoV‐2 antigens? Are people with MOGAD more "immunoreactive," and are their immune systems likely to develop antibodies against SARS‐CoV‐2 more "diligently"? Do they share any genetic predisposition backdrop with the few who develop MOGAD after COVID‐19 vaccination and in whom the close temporal association between with vaccination and MOGAD onset meets World Health Organization causality criteria for possible causation? [6] How would this relate to cases of neuromyelitis optica spectrum disorder that are aquaporin 4 IgG positive after COVID‐19 [7]?

There is merit in publishing negative studies. Sound scientific information can pertinently inform scientific judgement and further study, even if the results do not have the glamour of a "positive results" study. Multicentric data with enough subjects are paramount in rare diseases, and this study offers some data helping to design those studies.

## CONFLICT OF INTEREST

Neither of the authors has any conflict of interest to disclose.

## AUTHOR CONTRIBUTIONS

**Radu Tanasescu:** Conceptualization (equal), writing–original draft (equal), writing–review & editing (equal). **Markus Reindl:** Conceptualization (equal), writing–original draft (equal), writing–review & editing (equal).

## Data Availability

Not applicable.
